# Role of ENPP1 on Adipocyte Maturation

**DOI:** 10.1371/journal.pone.0000882

**Published:** 2007-09-12

**Authors:** Jian Liang, Mingui Fu, Ester Ciociola, Manisha Chandalia, Nicola Abate

**Affiliations:** 1 Department of Medicine, University of Texas Southwestern Medical Center, Dallas, Texas, United States of America; 2 Center for Human Nutrition, University of Texas Southwestern Medical Center, Dallas, Texas, United States of America; 3 Department of Molecular Biology and Microbiology, University of Central Florida, Orlando, Florida, United States of America; University of California at Los Angeles, United States of America

## Abstract

**Background:**

It is recognized that the ability of adipose tissue to expand in response to energy excess, i.e. adipocyte maturation, is important in determining systemic abnormalities in glucose and lipid metabolism. Ectonucleotide pyrophosphatase phosphodiesterase 1 (ENPP1, also known as PC-1) has been recently reported to be involved in the pathogenesis of insulin resistance and related diseases. However, its role on adipose tissue physiology as a mechanism of systemic insulin resistance is not understood. This study was performed to evaluate whether ENPP1 is regulated during adipogenesis and whether over-expression in adipocytes can affect adipocyte maturation, a potential novel mechanism of ENPP1-related insulin resistance.

**Methodology/Principal Findings:**

ENPP1 expression was found down-regulated during 3T3-L1 maturation, and over-expression of human ENPP1 in 3T3-L1 (pQCXIP-ENPP1 vector) resulted in adipocyte insulin resistance and in defective adipocyte maturation. Adipocyte maturation was more efficient in mesenchymal embryonal cells from ENPP1 knockout mice than from wild-type.

**Conclusions:**

We identify ENPP1 as a novel mechanism of defective adipocyte maturation. This mechanism could contribute to the pathogenesis of insulin resistance in absence of obesity.

## Introduction

The primary function of adipose tissue is to store energy in the form of triglycerides during periods of excessive energy intake. Adipocyte hypertrophy and hyperplasia are physiologic responses to energy excess that, if protracted, determine onset of obesity. This process often associates with abnormal adipose tissue function so that further triglyceride storage becomes inefficient. Consequent changes in plasma adipokine concentrations have the effect of modifying glucose and lipid metabolism in various tissues and organs [1]. This contributes to the clustering of metabolic abnormalities typically found in patients with the metabolic syndrome [2]. One important aspect of adipose tissue dysfunction is increased fatty acid spillover in the bloodstream [3], a condition that negatively impacts insulin-mediated glucose disposal in skeletal muscle [4]. This results in susceptibility to insulin resistance and its associated complications [5].

It has become increasingly recognized that the ability of adipose tissue to expand in response to energy excess, i.e. adipocyte maturation, is important in determining systemic abnormalities in glucose and lipid metabolism. For example, patients with lipodystrophy are unable to store even small amount of triglycerides due to either total or partial absence of adipose tissue [6]. Excessive caloric intake in these patients translates into high plasma non-esterified fatty acids (NEFA), increased triglyceride storage in lean tissues, severe insulin resistance and type 2 diabetes. This condition has been observed to a lesser degree also in the so called “metabolically obese lean persons” [7], [8]. It is possible that metabolically obese lean persons are unable to respond to caloric excess by increasing adipocyte maturation. Defective adipocyte maturation would then lead to excessive plasma NEFA and insulin resistance at lower body mass index (BMI). On the other hand, adipocyte maturation induced by thiazolindiones often resolves abnormalities in lipid and glucose metabolism. Therefore, understanding the role played by mediators of adipocyte maturation could provide important insight into the pathogenesis of insulin resistance in the absence of overt obesity.

It has been previously reported that the transmembrane glycoprotein ectonucleotide pyrophosphatase phosphodiesterase 1 (ENPP1; also known as plasma cell membrane glycoprotein PC-1) could be mechanistically linked to insulin resistance [9]. *ENPP1* interacts with the insulin receptor and inhibits subsequent signaling by decreasing its [beta] -subunit autophosphorylation [10]–[11]. We have found that *ENPP1 K121Q*, a common genetic polymorphism that determines increased *ENPP1*-insulin receptor interaction, can be associated with insulin resistance in absence of obesity [12]. In fact, we have also observed that *ENPP1 K121Q* is associated with lower BMI in a recessive model [13]. Since *ENPP1* is abundantly expressed in adipose tissue [14] and, since insulin plays a role on adipocyte maturation [15], the possibility of *ENPP1*-mediated defect in adipose tissue ability to expand in the presence of caloric excess should be considered as a mechanism of systemic insulin resistance. In the present study we evaluated the role of *ENPP1* in determining adipocyte maturation defect.

## Methods

### Cell culture and differentiation

3T3-L1 preadipocytes were cultured in Dulbecco's modified Eagle's medium (DMEM) supplemented with 10% fetal bovine serum, 100 U/ml penicillin, 100 µg/ml streptomycin in a 5% CO_2_ humidified atmosphere and allowed to reach confluence. Differentiation of two-day post-confluent preadipocytes was initiated with 5 µg/ml insulin, 1 M dexamethasone and 0.5 mM 3-iso-butyl-1-methylxanthine in DMEM supplemented with 10% fetal bovine serum. The culture medium was replaced every 48 h with DMEM supplemented with 10% fetal bovine serum and 5 µg/ml insulin.

### ENPP1 transfection

Human *ENPP1 K121K* and *ENPP1 K121Q* were transfected in 3T3-L1 using a *pQCXIP-ENPP1* vector. Both vector and *ENPP1* cells were then exposed to puromycin (2.5 µg/mL) for 7 days. Contaminated cells were eliminated.

### Oil Red O staining

Cells were washed twice with PBS and fixed with 10% formalin in PBS for 15 min. After two washes in PBS, cells were stained for at least 1 h in freshly diluted Oil Red O solution (six parts Oil Red O stock solution and four parts H_2_O; Oil Red O stock solution is 0.5% Oil Red O in isopropanol). The stain was then removed and the cells were washed twice with water, with or without counterstain (0.25% giemsa for 15 min) and then photographed.

### Triglyceride quantification in cultured cells

When cells in 96-well plates were 100% confluent, the culture supernatant was removed and each well rinsed with 200 ul of phosphate-buffered saline (PBS). Each well was filled with 200 ul of room temperature PBS. 5 ul of Adipored reagent (Cambrex Bioscience Walkersville, Inc.) were added to each well of the plate. Fluorescence with excitation at 485 nm and emission at 572 nm was measured to calculate triglyceride content.

### Cell death measurement

Cell death ratio was quantified by trypan blue assay. After washing with PBS, cell cultures were immediately stained with 0.4% trypan blue for 10 min, fixed with formalin, and rinsed with physiological saline. Unstained cells were regarded as viable and stained cells were regarded as dead. The viability of the cultures was calculated as the percentage ratio of the number of unstained cells relative to the total cells counted. Over 200 cells per cover slip were randomly counted.

### Glucose uptake Assay

3T3-L1 adipocytes were seeded in 6-well plates. Cells were serum starved for 3 h before the assay. Cells were then washed twice with KRPH buffer (5 mM Na_2_HPO_4_, 20 mM HEPES, pH 7.4, 1 mM MgSO_4_, 1 mM CaCl_2_, 136 mM NaCl, 4.7 mM KCl, 0.1% [wt/vol] BSA) and stimulated with 100 nM insulin or left untreated for 30 min. Glucose uptake was measured by incubation with 0.1 mM 2-deoxyglucose containing 1 µCi/ml 2-deoxy-d-glucose, [U-^14^C] at 4°C for 5 min. Transport was terminated by washing the cells three times with ice-cold PBS. Cells were solubilized with 0.2 mol/L NaOH, and the radioactivity was detected by scintillation counting. Nonspecific deoxyglucose uptake was measured in the presence of 20 µM cytochalasin B and was subtracted from each determination to obtain specific uptake.

### Immunoblot Analysis

Tissue was homogenized with a Potter-Elvehjem pestle in Cell Lysis Buffer (Cell Signaling Technology, Danvers, MA). Proteins were separated by SDS-PAGE, transferred to a nitrocellulose membrane (Bio-Rad), and incubated overnight with the antibody. Phospho-Insulin Receptor antibody (Sigma Aldrich), Insulin Receptor antibody (BD Bioscience, San Jose, CA), and human ENPP1 antibody (Imgenex Corp. San Diego, CA) were commercially available.

### Murine Embrional Fibroblasts (MEF) preparation

Embrional cells (EF) cells were isolated from *ENPP1* knockout (KO) mice embryos [16] at day 13 or 14 of gestation. Isolated embryos were place into dish with PBS and dissected to remove the head and soft tissue (liver, intestine, kidney, lung, heart). Embryo carcasses were placed into 10 cm dish and minced into fine pieces in trypsin/EDTA. Cell suspension was placed into T165 flask in EF medium and frozen after reaching confluence.

### mRNA Quantification

Total RNA was isolated from frozen tissues using RNA STAT-60 (Tel-Test, Friendswood, TX). Genomic DNA was removed from the total RNA preparations using DNAse 1 (DNA Free, Ambion). RNA from each sample was diluted to 5µng/µl and 100 ng RNA reverse transcribed in a 100 µl reaction using random hexamers (TaqMan Reverse Transcription kit, Applied Biosystems). To produce cDNA for standard curves, RNA from each of the control samples was pooled and serially diluted before cDNA synthesis. Each PCR reaction contained 2 µl cDNA, 150 nM each of forward and reverse primers, and 5 µl SYBR Green Universal PCR Master Mix (Applied Biosystems). Thermal cycling and data collection were performed using the ABI Prism 7900HT instrument (Applied Biosystems). Data were analyzed using SDS v2.2 software (Applied Biosystems). Relative quantification of gene expression was by the comparative C_T_ method (User Bulletin #2, Applied Biosystems). 18S mRNA was the endogenous control for total RNA content. Primers were designed using Primer Express v2.0 (Applied Biosystems) and synthesized by Integrated DNA Technologies. Amplification efficiency of each primer set was determined by analyzing the slope of the standard curve (User Bulletin #2, Applied Biosystems).

### Statistics

**Figure 1:**
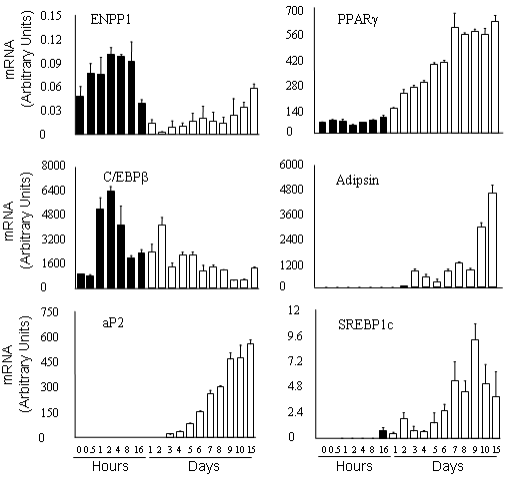
Expression of ENPP1 decreases in the early maturation phase of 3T3-L1 into adipocytes. 3T3-L1 cells were induced to differentiate into adipocytes by desamethasone, 3-iso-butyl-1-methylxanthine and insulin (DMI). The RNA was isolated at different time points, as indicated. Data show real time PCR for expression of ENPP1 and other genes involved in adipocyte maturation. Mean and SD from 3 experiments are presented in arbitrary units after correction for 18S expression. One-way ANOVA p-value for multiple group comparison was <0.05 for each of the gene expression data shown in figure. Pre-adipocyte are shown as dark bars, mature adipocyte are shown as open bars

Data are presented as mean±SD and were analyzed by 2-tailed Student's t test. One-way ANOVA was performed for multiple independent group comparisons (figure 1). Statistical analysis was performed using SAS version 8.02 (SAS Institute, Cary, NC).

## Results

Studies in 3T3-L1 showed that *ENPP1* expression is decreased during adipogenesis. Figure 1 shows the results on changes in *ENPP1* gene expression during the differentiation process of 3T3-L1 cells. We used quantitative PCR analysis in 3T3-L1 cells exposed to differentiation media containing dexamethasone, 3-iso-butyl-1-methylxanthine and insulin (DMI). *ENPP1* expression was significantly increased during the first 16 hrs of DMI-induced adipogenesis. However, its expression significantly decreased after the first day of adipocyte differentiation. This was associated with similar changes in expression of *C/EBP-β* a factor known to play a central role in adipocyte differentiation [17]. Gradual adipocyte maturation was coupled with increased expression of *PPAR-Υ, adipsin, aP2, SREBP1c*, genes that are also known to be regulated during the adipocyte differentiation process [18], [19].

**Figure 2:**
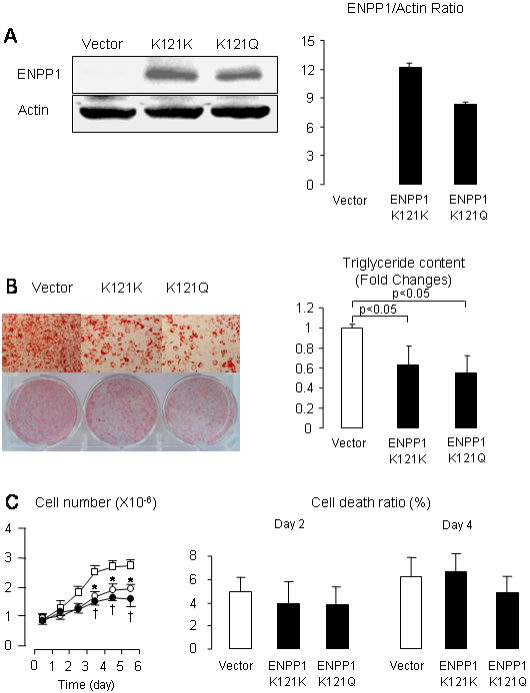
Human ENPP1 over-expression inhibits DMI-induced adipocyte maturation in 3T3-L1 cells. Human ENPP1 was over-expressed in 3T3-L1 using retrovirus transfection with CMV promoter. Panel A. ENPP1 protein content in cells containing vector only and in cells over-expressing human ENPP1 K121K or K121Q. ENPP1 was identified at 130 kDa using antibody from Imegex Corp. (San Diego, CA). Panel B. Oil-red-O stained 3T3-L1 cells after induction of adipogenesis. Cells were 100% confluent at the time of assay. Cellular triglyceride content is presented as fold changes from the triglyceride content of cells containing vector. Results are reported as mean and SD from measurements performed three times (8 wells measured in each experiment). Panel C. Cells were plated on 6-well plate and induced to differentiation with DMI. At different days, as indicated, the cell number was counted. Results are reported as mean and SD from measurements repeated three times (3 wells measured in each experiment). Open squares represent the data for the vector 3T3-L1. Open circles represent the data for 3T3-L1 over-expressing human ENPP1 K121K. Dark circles represent the data for 3T3-L1 over-expressing human ENPP1 K121Q. Cell death was measured at day 2 and day 4 of differentiation with DMI. Results are shown as mean and SD of percentage of dead cells relative to total cells. Measurements were performed for number of viable and dead cells in three wells for each cell type. These experiments were repeated 3 times. * p<0.05 for t-test between 3T3-L1 over-expressing human ENPP1 K121K and the vector group. † p<0.05 for t-test between 3T3-L1 over-expressing human ENPP1 K121Q and the vector group.

The coordinated changes in expression of *ENPP1* and adipogenetic molecules suggest a role of *ENPP1* in adipocyte maturation. To explore this possibility we generated 3T3-L1 over-expressing both human *ENPP1 K121K* and *ENPP1 K121Q*, using a pQCXIP-ENPP1 vector. As shown in Figure 2, antibody against *ENPP1* could detect high protein content in the cells over-expressing both forms of *ENPP1* but not in the vector cells. Maturation of adipocytes was decreased in 3T3-L1 over-expressing both types of *ENPP1*. Cells containing either K121K or K121Q genotypes had significant reduction in triglyceride content, as compared to the vector cells. Cell growth was significantly decreased in 3T3-L1 over-expressing human *ENPP1 K121K* and *K121Q*. On the other hand, cell death was not found to be different in the three different cell lines.

**Figure 3:**
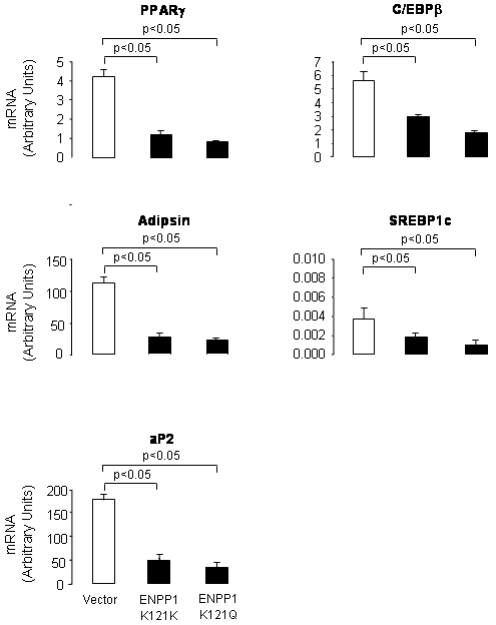
Human ENPP1 K121K and K121Q over-expression induce changes in the expression of genes present of 3T3-L1 induced to maturation. The expression levels of several adipogenic markers in vector only, in ENPP1 K121K and ENPP1 K121Q stable cell lines 10 days after DMI induction were measured by QPCR and normalized by the level of 18S. Experiments were repeated 3 times. The average values and SD are shown for the vector (open bars) and he transfected cells (dark bars).

To evaluate whether the inhibitory effects of *ENNP1* over-expression in 3T3-L1 cells associates also with a change in expression of genes involved in adipogenesis, we measured gene expression in the three cell lines, using QPCR approach. As shown in figure 3, over-expression of *ENPP1 K121K* or *K121Q* was associated with decreased expression of *PPAR-Υ, C/EBP-β, adipsin, SREBP1c,* and *aP2* at 10 days of DMI-induced differentiation.

**Figure 4:**
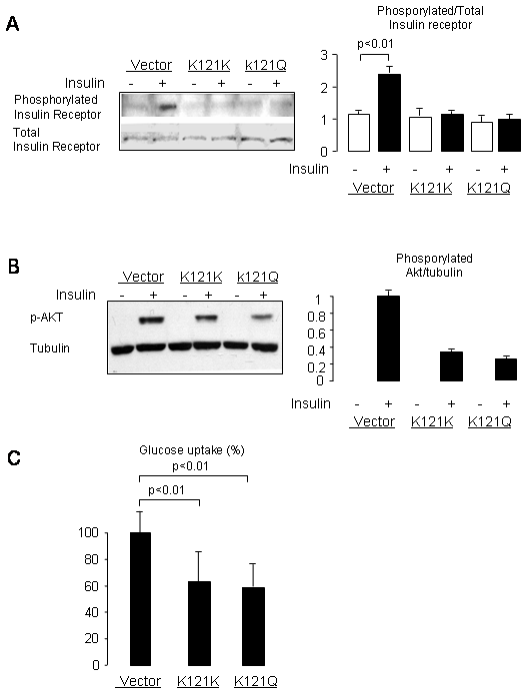
Human ENPP1 K121K and K121Q over-expression induce insulin resistance in 3T3-L1 cells. Panels A and B-Cells were treated with 5 µg/ml of insulin for 15 minutes. Total and phosporylated insulin receptor Akt were determined by Western Blot. Results are shown as bar graph of the ratio between phosphorylated and total insulin receptor. Phosphorylated Akt was determined by western blot. The quantitative values of the bands are shown beside the western blot image for 3 separate experiments. Panel C-3T3-L1 adipocytes were seeded in 6-well plates. Cells were stimulated with 100 nM and glucose uptake measured by incubation with 0.1 mM 2-deoxyglucose containing 1 µCi/ml 2-deoxy-d-glucose, [U-^14^C] at 4°C for 5 min.

To verify whether *ENPP1* over-expression associates with defective activation of insulin receptor, we measured both total and phosphorylated insulin receptor in vector cells and in cells over-expressing the K121K and K121Q forms of *ENPP1* following insulin stimulation (5 µg/mL of insulin for 15 minutes). As expected, insulin receptor phosphorylation was significantly decreased in the cells over-expressing human ENPP1 (Figure 4, Panel A). Downstream insulin-mediated Akt phosphorylation was also decreased in cells over-expressing *ENPP1* (Figure 4, Panel B). Glucose uptake in the presence of insulin was decreased in cells over-expressing *ENPP1* (Figure 4, Panel C).

**Figure 5:**
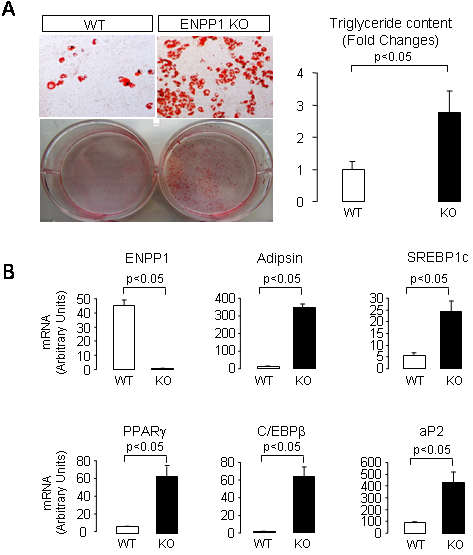
Adipocyte differentiation is enhanced in the absence of ENPP1. Panel A. Oil-red-O stained mouse embrionic fibroblasts (MEF) from ENPP1 knockout mice after induction of adipogenesis using stimulation with DMI. Cellular triglyceride content is presented as fold changes from the triglyceride content of cells containing vector. Results are reported as mean and SD from three experiments (8 wells were analyzed in each experiment). Panel B. The expression levels of several adipogenic markers in differentiated wild-type and ENPP1 knockout MEFs were measured by QPCR and normalized by the level of 18S. Experiments were repeated 3 times.

To further confirm a role of *ENPP1* regulation on adipogenesis, we studied mouse embryonic fibroblasts (MEF) of *ENPP1* knockout mice. As shown in Figure 5 (panel A), DMI-induced adipocyte maturation was more effective in MEF from *ENPP1* knockout mice than in MEF from wild-type mice. These effects on adipocyte maturation were seen in concomitance not only to an absent expression of *ENPP1* but also to significantly increased expression of adipsin, *SREBP1c, PPAR-Υ, C/EBP-β* and *aP2* gene expression in the *ENPP1* knockout adipocytes (Panel B of Figure 5).

## Discussion

The main finding of this study is that *ENPP1* expression is involved in regulation of adipocyte maturation, a mechanism that may contribute to systemic fatty acid and glucose metabolism maintenance during weight gain. We first found that *ENPP1* is highly regulated during adipogenesis. We also found that its increased interaction with the insulin receptor not only associates with defective cellular insulin signaling, as previously described [9], [10], but also with defective adipocyte maturation.

Previous investigations have suggested that *ENPP1* over-expression specifically induces defective insulin signaling in cells [20]. This effect has also been observed with the common *ENPP1 K121Q* genetic variant, and attributed to a stronger interaction between the non-catalytic domains of the mutated ENPP1 with the insulin receptor [11]. Consequent reduction in insulin signaling transduction from the α- to the β-subunit of the insulin receptor would reduce the ability of insulin to stimulate downstream signaling cascade. According to this view, *ENPP1* over-expression in liver and muscle, two main tissues involved in insulin-mediated regulation of glucose and lipid metabolism, determines insulin resistance and susceptibility to type 2 diabetes. Recent animal models over-expressing *ENPP1* in liver and in other tissues, including muscle, have shown insulin resistant phenotype [21], [22]. However, these animal models have not included *ENPP1* over-expression in adipose tissue. The possibility that *ENPP1*, would impact glucose and lipid metabolism through an effect on adipocyte function had not been previously evaluated. This seems to be of importance in light of the high expression of *ENPP1* in human adipose tissue [14] and in light of the role that adipose tissue function plays in the pathogenesis of systemic insulin resistance and type 2 diabetes. In addition, we have recently shown an interaction between *ENPP1 121Q* variant and BMI in humans in predicting insulin resistance [12] and type 2 diabetes [23]. In those studies we have suggested that lack of inclusion of this variable may contribute to the apparent discrepancy among published genetic association studies on *ENPP1 K121Q* and type 2 diabetes. Although previous studies in 3T3-L1 have failed to detect effects of *ENPP1* over-expression on cellular insulin sensitivity [24], our results show that *ENPP1* over-expression can impair insulin receptor activation in 3T3-L1 cells (figure 4). This apparent discrepancy could be related to the different approach used to obtain *ENPP1* over-expression in 3T3-L1. We used a pQCXIP-ENPP1 vector to obtain stable transfected cell lines, as opposed to the adenovirus system used in the study by Sakoda et al. [24].

A novel finding of our study is that *ENPP1* is highly regulated during adipogenesis. Although we did not address the mechanistic details of *ENPP1* regulation, we determined that *ENPP1* expression is coordinated with changes in expression of genes that are known to be involved in the adipocyte maturation progress (Figure 2) [17]–[19]. We also show that over-expression of *ENPP1* in transfected 3T3-L1 cells will affect maturation process in association with decreased insulin-mediated activation of insulin receptor and with insulin-mediated glucose uptake. Insulin increases the percentage of cells that differentiate, and also increases the amount of lipid accumulation in each fat cell [15]. Although other mechanisms mediating *ENPP1* effect on adipogenesis cannot be excluded, the possibility exists that interference with insulin receptor function plays a role in modulation of adipocyte maturation. Therefore, the results of our study support the new hypothesis that inheritance of the common genetic variant *ENPP1 K121Q*, a gain of function on the *ENPP1*-induced insulin receptor inhibition [10], would predispose individuals to decreased ability in forming mature adipocyte when storage of excessive energy intake is needed. As a consequence, decreased fatty acid uptake by adipocytes and/or excessive lipolysis could cause high plasma NEFA concentrations, hyperinsulinemia and defective insulin-mediated glucose utilization in skeletal muscle. *ENPP1 K121Q* has been previously associated with human obesity [14]. On the other hand, whether this association was related to coexisting confounding factors, such as diabetes, is not clear. In fact, other studies have suggested an association with decreased body mass index (BMI) [13], [25]. Clearly, mechanisms for adipogenesis and onset of obesity are complex, and alternative pathways can compensate for the inhibitory effects of *ENPP1* on adipogenesis. Regardless, the effects of increased *ENPP1* expression on adipocyte maturation could have a negative influence on the overall adipose tissue ability to compensate for excessive caloric intake and maintain normal systemic glucose and lipid metabolism.

The results of our study and the high frequency of *ENPP1 K121Q* in humans [26] support the view that among the various described mechanisms of insulin resistance mediated by defective adipogenesis, such as in lipodystrophy models [27], [28], in defective *PPAR-γ* function [29], and in Pref-1 overproduction [30], *ENPP1* expression and its K121Q variant are likely to explain a large portion of the “garden variety” insulin resistance we find in people at relatively low body mass index (BMI). ENPP1 K121Q could be an important determinant of genetic susceptibility to insulin resistance and may provide a useful clinical marker and a therapeutic target for insulin resistance, type 2 diabetes and cardiovascular disease.

In summary, the results of our studies support a role of increased *ENPP1* expression in the pathogenesis of insulin resistance in humans. In addition to previously proposed mechanisms of systemic insulin resistance mediated by effects of *ENPP1* on cellular insulin function in hepatocyte and myocyte, we suggest that its effect on glucose metabolism is, at least in part, mechanistically linked to *ENPP1*-induced inhibition of adipocyte maturation. Defective adipocyte maturation related to increased *ENPP1* function does not exclude the possibility of obesity [14]. However, it may contribute to earlier onset of insulin resistance and related morbidities even with modest body fat increase [12], [23].
